# Case report: Drug reaction with eosinophilia and systemic symptoms (DRESS)-induced hemophagocytic disorder

**DOI:** 10.3389/fphar.2022.1023522

**Published:** 2022-11-09

**Authors:** Eliza I. Pope, Hosanna Au, Deborah M. Levy, Ruud H. J. Verstegen

**Affiliations:** ^1^ Department of Paediatrics, University of Toronto, Toronto, ON, Canada; ^2^ Division of Paediatric Medicine, Department of Paediatrics, The Hospital for Sick Children, Toronto, ON, Canada; ^3^ Division of Rheumatology, Department of Paediatrics, The Hospital for Sick Children, Toronto, ON, Canada; ^4^ Division of Clinical Pharmacology and Toxicology, Department of Paediatrics, The Hospital for Sick Children, Toronto, ON, Canada

**Keywords:** DRESS (drug reaction with eosinophilia and systemic symptoms), antibiotics, drug reaction, hemophagocytosis, pediatrics, case report, hemophagocytic lymphohistiocytosis, macrophage activation syndrome

## Abstract

Hemophagocytic disorders are severe and life-threatening conditions that can be genetic in origin [i.e., primary hemophagocytic lymphohistiocytosis (HLH)] or result from infections (i.e., secondary hemophagocytic lymphohistiocytosis), rheumatologic disease [i.e., macrophage activation syndrome (MAS)], and less frequently immunodeficiency or metabolic disease. Although rare, drug-induced hemophagocytosis needs to be considered in the work-up as it requires specific management strategies. Most drug-induced hemophagocytic disorders are related to Drug Reaction with Eosinophilia and Systemic Symptoms (DRESS). We present the case of a 7-year-old girl who initially presented with fever, maculopapular rash, and unilateral lymphadenopathy, who went on to develop hemophagocytosis secondary to DRESS caused by prolonged combination treatment with amoxicillin/clavulanic acid and trimethoprim/sulfamethoxazole. This case illustrates the importance of considering adverse drug reactions in the evaluations of patients with a hemophagocytic process.

## Introduction

Hemophagocytic disorders represent a spectrum of conditions characterized by hyperinflammation, pancytopenia, and end-organ involvement. Although there is no consensus on the nomenclature, terminology is often based on the (suspected) etiology. Primary hemophagocytic lymphohistiocytosis (HLH) has a genetic origin, secondary HLH is generally related to infections, and macrophage activation syndrome (MAS) refers to an underlying autoimmune or autoinflammatory condition (Crayne and Cron, 2019). Less frequently, malignancies, immunodeficiencies, and metabolic disease can cause hemophagocytosis, which—although inconsistently—may also be referred to as secondary HLH. Drug-induced hemophagocytic disorders are presumed to be rare but are likely underdiagnosed.

We cared for a child presenting with a hemophagocytic disorder that was triggered by Drug Reaction with Eosinophilia and Systemic Symptoms (DRESS), a severe drug-induced multi-system inflammatory condition. Although cutaneous, hepatic, renal and respiratory involvement is most common, all other organs can be affected. Overall, DRESS has a high mortality rate of up to 10% (Husain et al., 2013a). Like hemophagocytic disorders, it also involves marked activation of T cells, as evidenced by elevated soluble CD25 levels (Kitcharoensakkul et al., 2012; Cho et al., 2017; Crayne and Cron, 2019). In addition, reactivation of herpes viruses (e.g., Epstein–Barr virus, cytomegalovirus, HHV-6, and HHV-7) is thought to contribute to the pathophysiology (Cho et al., 2017).

## Case summary

A 7-year-old girl with mild right hemiparetic cerebral palsy and a diagnosis of chronic Lyme disease was seen for prolonged fever. She had been well until 2 weeks prior when she developed a high-grade fever persisting for 5 days and a maculopapular rash on her trunk, which had spread to her face and lower limbs. The family sought medical attention locally, and her symptoms were felt to be consistent with parvovirus infection. She defervesced for 5 days but became febrile again. After 3 days of daily fevers up to 40°C, she was seen in our Emergency Department with non-bilious, non-bloody emesis. There was no history of other infectious symptoms, sick contacts, or travel. She had no known drug allergies, and her immunizations were up to date. She had been on several courses of antibiotics for chronic Lyme disease and had started a daily combination of amoxicillin/clavulanic acid and trimethoprim/sulfamethoxazole 4 weeks prior to her presentation. She had also taken a variety of naturopathic/homeopathic products for more than 6 months as part of the management of chronic Lyme disease.

**FIGURE 1:**
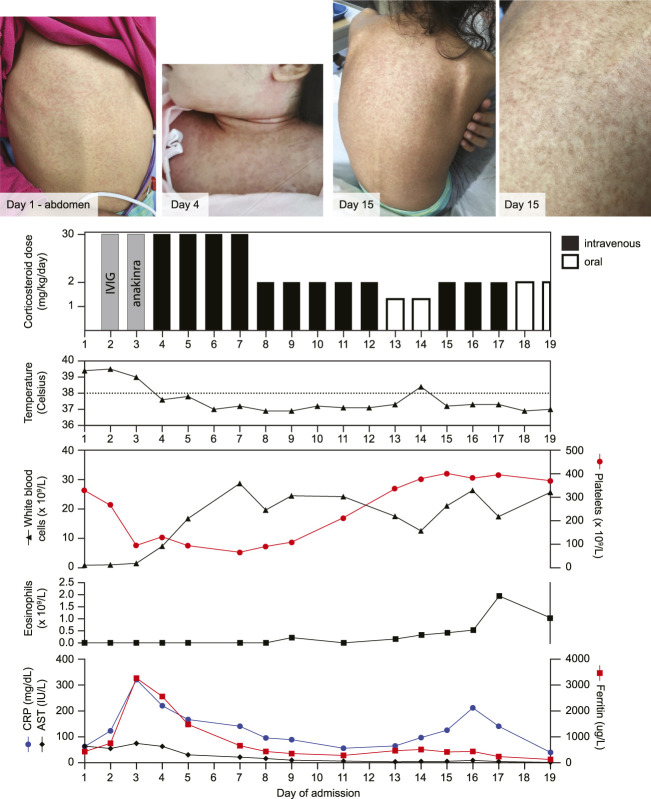
Evolution of the maculopapular rash alongside treatment timeline and laboratory investigations. Intravenous immunoglobulin (IVIG; 2 g/kg/dose) and anakinra (3.7 mg/kg/dose) were both given once. CRP: C-reactive protein, AST: aspartate aminotransferase.

On physical examination, she had a non-toxic appearance, with a diffuse non-desquamating maculopapular rash on her trunk and back with no involvement of the extremities (Figure 1). There was no facial edema, conjunctivitis, or mucosal involvement. There was bilateral cervical lymphadenopathy with one lymph node measuring more than 1.5 cm. In addition, she had diffusely tender axillary and inguinal lymphadenopathy. The remainder of the physical examination was non-contributory. At the time of presentation, laboratory investigations showed a normal hemoglobin and platelet count, leukopenia (0.88 × 10^9^/L), and an increased CRP (60 mg/L). She was COVID-19 PCR-negative.

On initial presentation to the Emergency Department, her home medications were discontinued, and she was started on intravenous broad-spectrum antibiotics. The presence of prolonged fever, rash, and cervical lymphadenopathy led to a presumed diagnosis of incomplete Kawasaki disease for which she received intravenous immunoglobulin (IVIG; 2 g/kg). An echocardiogram showed no coronary aneurysms. She was persistently febrile, and repeated laboratory investigations became concerning for a hemophagocytic disorder, as suggested by the presence of pancytopenia, systemic hyperinflammation with paradoxical decreased erythrocyte sedimentation rate (ESR), hyperferritinemia, transaminitis, and persistent coagulopathy despite three doses of vitamin K (Figure 1). A bone marrow biopsy was negative for malignancy and did not show hemophagocytosis, which is not required for the diagnosis of hemophagocytic disorders. A broad infectious work-up was negative for blood or urine bacterial infections, and enteric bacterial and viral infections, as well as adenovirus, CMV, EBV, HSV, HHV-6, parvovirus, and varicella in the blood. The HHV-7 blood PCR was positive. COVID serology was negative. A chest X-ray was normal.

She continued to deteriorate, requiring admission to the Pediatric Intensive Care Unit for hemodynamic instability with multiorgan dysfunction, evidenced by liver dysfunction and inflammatory ileus. Following the administration of IVIG, one dose of anakinra (3.7 mg/kg) was given to treat the hemophagocytic disorder. Subsequently, she was started on intravenous methylprednisolone pulse (30 mg/kg/dose) for four consecutive days, which resulted in a rapid clinical improvement. Given the timeline of her antibiotic exposure, she was suspected to have DRESS. Subsequently, she continued daily intravenous methylprednisolone and was switched to an oral prednisone taper. One day after starting the taper, there was a recurrence of a diffuse morbilliform rash and worsening inflammatory markers with marked eosinophilia (Figure 1), which was also consistent with DRESS. She was switched back to her initial dose of intravenous methylprednisolone, and within 2 days, her symptoms improved. Prednisone was tapered gradually over a 3-month period without any resurgence of symptoms. She developed hypertension which was treated with amlodipine. Twelve months after diagnosis, she was doing well and had not developed long-term sequelae related to DRESS.

## Discussion

We have described a patient with an unusual etiology of hemophagocytic disorder, which was initially attributed to an undefined rheumatologic condition. However, as the disease progressed, it became apparent that the patient’s condition was caused by DRESS. The diagnosis of DRESS was supported by fever, rash, cervical lymphadenopathy, eosinophilia, atypical lymphocytes, internal organ involvement (e.g., hepatic and gastrointestinal involvement), and human herpesvirus (HHV)-7 positivity. Both amoxicillin/clavulanic acid and trimethoprim/sulfamethoxazole have been reported as causes of DRESS. Furthermore, the onset of symptoms 4 weeks post-initiation of the oral antibiotics is consistent with the typical timeline seen in DRESS, where symptoms generally develop after 2–6 weeks of treatment (Husain et al., 2013a). The naturopathic and homeopathic products had been taken regularly for more than 6 months, making those unlikely culprits. Finally, the rapid increase in disease activity, following the initial steroid taper, has been reported in DRESS, and a slow taper is, therefore, recommended to prevent recurrence (Husain et al., 2013b).

This clinical presentation illustrates how DRESS can trigger a hemophagocytic process, which is important for physicians who care for children and adults during the initial workup and management of those conditions. First, the identification of DRESS should lead to the immediate discontinuation of the offending agent. Second, once DRESS is diagnosed, unnecessary investigations looking for other conditions related to hemophagocytic disorders may be avoided. Third, physicians should look for DRESS-specific complications. Virtually, all organs can be involved in this condition, but pancreatic and thyroid involvement is more likely to occur in DRESS compared to other causes of hemophagocytic disorders (Husain et al., 2013a). Long-term complications of DRESS may warrant further monitoring; in particular, patients are more likely to develop a variety of autoimmune conditions such as thyroid disease, type I diabetes, and systemic lupus erythematosus (Chen et al., 2013). Finally, without identification of DRESS and its trigger, there is a risk of recurrence due to inadvertent re-exposure.

Although the development of a hemophagocytic process in the context of DRESS is not new (Picard et al., 2013; Korbi et al., 2015; Penel-Page et al., 2017; Rioualen et al., 2017; Carter Febres et al., 2021), this complication is significantly underdiagnosed. Cohort studies with prospectively collected data on this complication are lacking, but as cytopenia is present in 10–30% of DRESS patients (Choudhary et al., 2013), it is likely that at least a proportion of these patients has concurrent hemophagocytosis. In most institutions, patients with DRESS are managed by general pediatricians or internists, who may have limited experience with this infrequent condition. Although cessation of the offending agent is the primary treatment for DRESS, it may not halt the underlying cytokine storm related to a hemophagocytic disorder. Additional treatment in the form of corticosteroids and potentially additional immunosuppressive medications (i.e., cyclosporine and IL-1 blockade) may, therefore, be necessary to prevent the significant morbidity and mortality associated with this complication (Husain et al., 2013b; Nguyen et al., 2020).

It is important for clinicians to realize that eosinophilia is absent in about one-third of DRESS patients (Cacoub et al., 2011). In particular, those affected by DRESS-associated hemophagocytic disorders are more likely to have a normal eosinophil count, due to their leukopenia. This was also seen in our patient, who initially lacked eosinophilia.

Given the overwhelming release of pro-inflammatory mediators such as IFNγ, interleukin (IL)-1, IL-6, and tumor necrosis factor (Cho et al., 2017), it is not difficult to understand that DRESS can trigger the development of a hemophagocytic disorder. However, alternative mechanisms of drug-induced hemophagocytic disorders are present that may need to be considered for individual patients. For example, other drug-induced inflammatory conditions, such as drug-induced lupus erythematosus, may be complicated by hemophagocytic disorders (Leith and Savage, 102019).

Although amoxicillin/clavulanic acid and trimethoprim/sulfamethoxazole are commonly used oral antibiotics, their association with DRESS is not as strong as some other medications (e.g., carbamazepine, sulfonamides, and piperacillin/tazobactam). However, the long duration of treatment predisposed this patient to develop this unusual reaction. We, therefore, urge clinicians to remain judicious and thoughtful when prescribing even commonly used oral antibiotics for a prolonged duration, recognizing the potential clinical implications. Future research could focus on the identification of at-risk populations, for example, by studying the association between HLA types and the development of DRESS, which may lead to pre-treatment testing and prevention of this severe adverse drug reaction.

## Data Availability

The original contributions presented in the study are included in the article/supplementary material, further inquiries can be directed to the corresponding author.

## Abbreviations

MAS, macrophage activation syndrome; HLH, hemophagocytic lymphohistiocytosis
